# Impact of *Amirthalingamia macracantha* Larvae on Nile Tilapia (*Oreochromis niloticus*): A Morpho-Histopathological Perspective

**DOI:** 10.3390/ani15162334

**Published:** 2025-08-09

**Authors:** Ebtsam Sayed Hassan Abdallah, Mahmoud Mostafa Mahmoud, Hanan Hassan Abdel-Hafeez, Marco Albano, Gioele Capillo, Asmaa Mohamed Metwally, Sultan Mohammed Areshi, Dalal Alardan, Yosra M. I. El Sherry

**Affiliations:** 1 Department of Aquatic Animal Medicine and Management, Faculty of Veterinary Medicine, Assiut University, Assiut 71529, Egypt; ebtsamsayed@aun.edu.eg (E.S.H.A.); mahmoud88@aun.edu.eg (M.M.M.); 2 Department of Cell and Tissue, Faculty of Veterinary Medicine, Assiut University, Assiut 71516, Egypt; hhnnzz91@aun.edu.eg; 3 Department of Veterinary Sciences, University of Messina, Polo Universitario Dell’Annunziata, 98168 Messina, Italy; 4 Department of Chemical, Biological, Pharmaceutical and Environmental Sciences, University of Messina, Viale Ferdinando Stagno D’Alcontres 31, 98166 Messina, Italy; gcapillo@unime.it; 5 Sea in Health and Life Srl, Capo Peloro, 98164 Messina, Italy; 6 Department of Parasitology, Faculty of Veterinary Medicine, South Valley University, Qena 83523, Egypt; 7 Biology Department, Science College, Jazan University, Jazan 45142, Saudi Arabia; sareshi@jazanu.edu.sa; 8 Department of Biology, College of Science, University of Hail, Hail 2440, Saudi Arabia; d.alardan@uoh.edu.sa; 9 Department of Fish Diseases, Faculty of Veterinary Medicine, Aswan University, Sahari, Airport Way, Aswan 81528, Egypt; yosramohamed@vet.aswu.edu.eg

**Keywords:** teleost, Egypt, fish organs, infestation parasite morphology, histopathological study

## Abstract

*Amirthalingamia macracantha* (Joyeux and Baer, 1935) larvae, a member of the Gryporhynchidae family (Cestoda: *Cyclophyllidea*), are commonly found in a variety of African fish species, including Nile tilapia (*Oreochromis niloticus*, Linnaeus, 1758). Nonetheless, little is known regarding their existence in native Egyptian fish species. Two hundred *O. niloticus* were examined in the current investigation, using standard parasitological techniques to detect the presence of these larvae in different fish organs. According to this study’s findings, *A. macracantha* larvae were found in 35% of the microscopically examined samples as red nodules, attaching to the serosa of both the anterior and posterior sections of the intestine and stomach, respectively. The mean abundance of the infection was 3.5 ± 0.05 (CI 95% 3.4–3.5), and its mean intensity was 10 ± 1.7 (CI 95% 8.2–11.7). The existence of two rows of twenty rostellar hooks of varying lengths is the main characteristic of the parasite. The upper row consisted of four large hooks and six smaller ones, while the lower row contained smaller hooks of consistent length. According to histopathological analysis, the larvae with a hook-and-spiny rostellum were affixed to the stomach serosa of *O. niloticus*. Additionally, there was a notable presence of degranulated mast cells and lymphocytes of varying sizes within the stomach’s lamina propria, submucosa, and blood vessels, as well as a loss of gastric epithelium, fibrosis, and enlargement of submucosal lymph vessels. This study offers a succinct description of this larval stage, representing the first reported occurrence of its presence in Egyptian *O. niloticus*.

## Simple Summary

One of the most widely farmed fish species globally is Nile tilapia, *Oreochromis niloticus*. *Amirthalingamia macracantha* (Joyeux and Baer, 1935) larvae lead to high mortality, especially in juvenile fish, due to critical pathological alterations in main organs such as the intestine, liver, and stomach. Since there have not been any previous records of *A. macracantha* (Joyeux and Baer, 1935) larvae in Assiut Province, Egypt, this study intends to investigate the presence of *A. macracantha* larvae in wild *O. niloticus*. The larvae of *A. macracantha* are found in the serosa of both the anterior and posterior regions of the intestine and stomach, producing red nodules. One of the main characteristics of the parasite is the presence of two rows of twenty rostellar hooks that vary in length. There are four large hooks and six smaller ones in the upper row, while the lower row features smaller hooks that are of similar lengths. These distinctive characteristics help to identify the parasite.

## 1. Introduction

Nile tilapia (*Oreochromis niloticus*) is one of the most globally farmed fish species, accounting for 8.3 per cent of global finfish production [1]. *O. niloticus* is known for its high environmental adaptability, able to thrive in a wide range of aquatic conditions from tropical to temperate environments. This fast-growing freshwater species, autochthonous to North Africa, is globally distributed due to its aquaculture uses for over four decades, representing an essential commercial resource for many developing countries [2]. Moreover, it represents an important nutritional resource for the communities of African and Asian countries [3]. The large use of this teleost in human commercial activities led to its use in scientific research, with dedicated programs for genetic improvement, breeding techniques, and feeding strategies [4,5]. Despite its high resistance to diseases and stressful conditions, studies on pathologies and parasites that could affect its performance in nature and aquaculture are a topic of interest in many research projects worldwide [6,7].

Tilapia is more commonly referred to as the second intermediate or paratenic host of metacestodes [8]. Most of these species belong to the family Gryporhynchidae (Cyclophyllidea), using copepods as their first intermediate host and fish-eating birds as their final host [9].

The bodies of Gryporhynchidae tapeworms are small to medium in size, reaching a maximum length of 10 cm. The scolex surface has four circular suckers, with a retractable proboscis in the rostellum. There are two rows of hooks with a handle, guard, and blade on each hook. The strobila contains three to several hermaphrodite proglottids, with the characteristics of the genital system being crucial for adult classification [10].

Gryporhynchid larvae have been found encysted or free-moving. Except for the non-encysted *Amirthalingamia macracantha*, which can grow up to 17 mm, they are typically small (1–2 mm). Some species exhibit strict host and site specificity, while others are found in a variety of fish hosts [11], parasitizing internal organs such as the liver, mesentery, gallbladder, and the walls of the stomach and intestine [8,11]. When larval stages attack vital organs like the liver, they can cause serious harm to farmed tilapia, particularly young fish [8]. The presence of a scolex with two rows of rostellar hooks and four suckers distinguishes the gryporhynchid larval stage [12].

The parasite has been detected in wild and cultured *O. niloticus* in many African countries, such as Sudan, Uganda, Ethiopia, and Kenya [5,13,14,15,16,17]. However, there is not much concern about this larval stage in Egypt. Therefore, this study aimed to assess morphometric and morphological characteristics of this larval stage and its impact on the stomach and intestine of wild *O. niloticus* captured in Assiut Province, Upper Egypt. Furthermore, mean intensity, mean abundance, worm burden, and prevalence of these larvae were computed.

## 2. Materials and Methods

### 2.1. Study Area and Fish Collection

**Figure 1:**
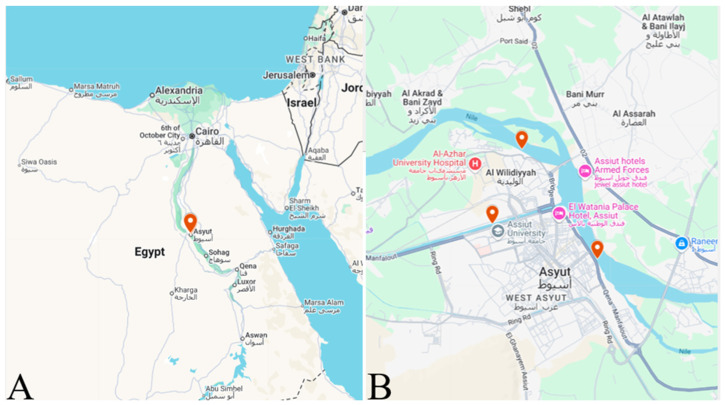
(**A**) Map of Assiut Governorate (marked in red) in Egypt; (**B**) various sampling collection sites within Assiut Governorate (https://www.google.com/maps/d/edit?mid=1sSrcs_5YOK9RGp2vDi3woxSEhc-Va70&usp=sharing, accessed on 31 July 2025).

The fish used in the present study were handled and treated strictly following procedures outlined in the Guide for the Use of Experimental Animals by the Welfare Committee of the Faculty of Veterinary Medicine, at Assiut University, in Assiut, Egypt (Code No. 06/2024/0165). Two hundred live wild *O. niloticus* were selected based on criteria established by Shvydka and colleagues [18]. The fish had an average body weight of 33.5 ± 6.7 g, a total length of 12.5 ± 0.7 cm, and a standard length of 10.0 ± 0.7 cm. They were collected alive from the River Nile in Assiut City, located in southern Egypt (latitude 27°10′51.46″ N, longitude 31°11′1.25″ E, as shown in Figure 1) from three different sampling sites by local fishermen. They were then transported alive to the university laboratory for parasitological examination.

The fish were promptly transported to the Aquatic Laboratory at the Faculty of Veterinary Medicine, Assiut University, for examination. They were assessed for any visible clinical signs and postmortem lesions, following the methods outlined by Eissa [19] and Noga [20]. Before the examination, the fish were euthanized using 200 µL of clove oil per liter of tank water [21] as described in previous studies [22,23,24,25,26].

### 2.2. Parasitological Examination

For each fish specimen, the stomach and the intestine were examined, searching for the encysted larval stage. After the dissection, the infected parts were placed in a physiological saline solution following the methods of Abdallah and colleagues [27], as well as Thabit and Abdallah [25]. The length and width of the parasites were measured to the nearest micrometer (µm), photographed using a Leica microscope (Leica DM 1000, Wetzlar, Germany), and analyzed microscopically. The prevalence (number of infected fish/total number of examined fish × 100), mean intensity (total number of parasites collected/total number of infected fish), and mean abundance (total number of parasites collected/total number of examined fish) of the parasites were calculated according to Bush et al. [28]. The confidence interval was calculated according to Reiczigel et al. [29]. The fish condition factor of the infected fish was calculated using the formula developed by Pauly [30], where the condition factor (K) = 100 × W/L^3^, with W representing weight (g) and L representing total length (cm).

### 2.3. Histopathological Examination

#### 2.3.1. Paraffin Processing Technique

Fresh specimens from the stomach of the fish in the infected groups were collected and fixed in Wrobel–Moustafa Fixative [31]. The tissues were dehydrated in a graded alcohol series, cleared with xylene, embedded in paraffin wax, sectioned at 4–5 μm thickness, and stained with hematoxylin and eosin and Picrosirius red [32,33], PAS (periodic acid–Schiff)–Harris hematoxylin, and methylene blue for histopathological examination using light microscopy [34,35,36].

#### 2.3.2. Quantitative Morphometrical Analysis

Morphometric measurements were conducted utilizing an image analysis system. The quantity of inflammatory cells was assessed across ten slides for each specimen block. The cell count was approximated utilizing the mean and standard deviation (SD). Furthermore, by the procedures outlined by Abdel Hakeem et al. [37], the percentage of fibrous tissue was determined by staining slides with Picrosirius red.

### 2.4. Statistical Analysis

The data was carefully entered into a spreadsheet in Microsoft Excel 2010 and subsequently analyzed using Prism^®^ 8 software (version 8.4.3) programmed onto GraphPad (Boston, MA, USA). A *t*-test was used to determine the significance of the condition factor. Additionally, a two-way ANOVA (Tukey test) was employed to analyze the number of inflammatory cells in the lamina propria and submucosa layers compared to the healthy control group. Each result represents the mean of three replicates ± the standard error of the mean (SEM) value.

## 3. Results

### 3.1. Parasitological Examination

**Figure 2:**
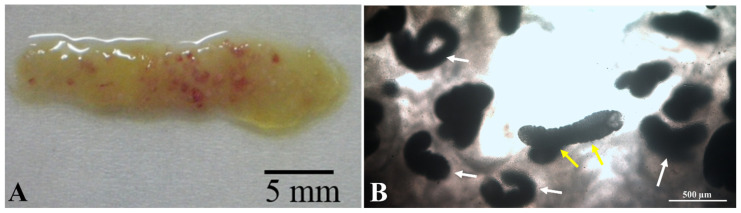
The intestine of Nile tilapia (*Oreochromis niloticus*) infected with *Amirthalingamia macracantha* larval stage cysts shows distinct characteristics. (**A**) Macroscopically, they appear as small red nodules. (**B**) Microscopically, numerous *A. macracantha* larval stages are seen encysted in the intestinal wall (indicated by white arrows), with some free parasites found outside the cyst (indicated by yellow arrows) in a wet mount.

Cysts of the *A. macracantha* larval stage were discovered in the mucosa of the posterior region of the stomach and the anterior region of *O. niloticus* intestinal mucosa, indicating that this host is the second intermediate host (Figure 2). Out of the 200 fish tested, 70 individuals were found to be infected with this larval stage, resulting in a prevalence of 35 per cent. The number of *A. macracantha* cysts per fish varied from one to forty-five, with a mean intensity of 10 ± 1.7 (CI 95% 8.2–11.7) and a mean abundance of 3.5 ± 0.05 (CI 95% 3.4–3.5). The condition factor of the infected fish was 1.67 ± 0.03 (CI 95% 1.64–1.69), which was significantly different (*p* value < 0.0001; *t*-test) from non-infected fish, whose condition factor was 2.1 ± 0.05 (CI 95% 2.05–2.15). To the naked eye, the parasite cysts appeared as small red nodules (Figure 2A).

**Figure 3:**
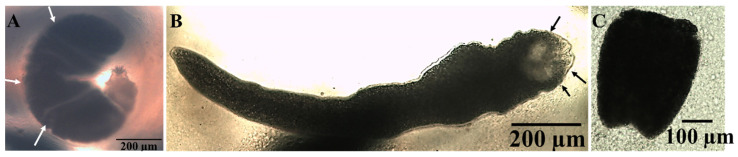
A single *Amirthalingamia macracantha* larval stage is observed in a wet mount from the intestine of *Oreochromis niloticus*. It displays the following microscopic features: (**A**) an encysted larval stage inside a cyst (white arrow), (**B**) a free stretched parasite with a folded anterior body part and an inverted rostellum in the scolex (black arrows), and (**C**) a contracted free parasite.

Microscopically, the cyst diameter was 647.8 ± 12.5 × 534.3 ± 24.2 µm (Figure 2B and Figure 3A). The body of the larval stage was folded (Figure 3B), with an average overall length including scolex and neck of 1228.5 ± 34.7 µm, a body length without scolex of 903.1 ± 31.8 µm, and a width of 312.6 ± 36.0 µm, respectively. In contrast, the contracted free stage has dimensions of 390.0 ± 15 µm in length and 312.5 ± 2.1 µm in width, appearing much shorter in length (Figure 3C).

**Figure 4:**
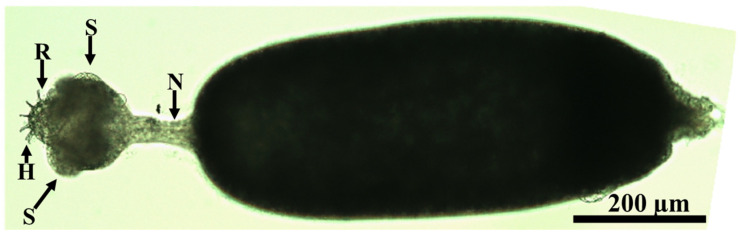
*Amirthalingamia macracantha* larval stage isolated from the intestine of *Oreochromis niloticus*. It has a stretched neck (N) and an everted rostellum (R) of the scolex carrying four suckers (S) and two rows of hooks (H).

**Figure 5:**
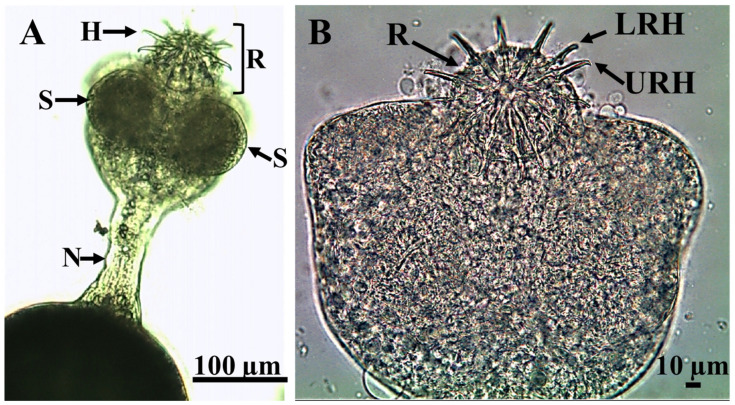
*Amirthalingamia macracantha* larval stage isolated from the intestine of *Oreochromis niloticus* displaying (**A**) a stretched neck (N) and an everted rostellum (R) with two rows of hooks (H) and two suckers (S); (**B**) a higher magnification of the scolex showing the upper row of ten hooks (URH) attached to the rostellum (R) and the lower row of hooks (LRH).

**Figure 6:**
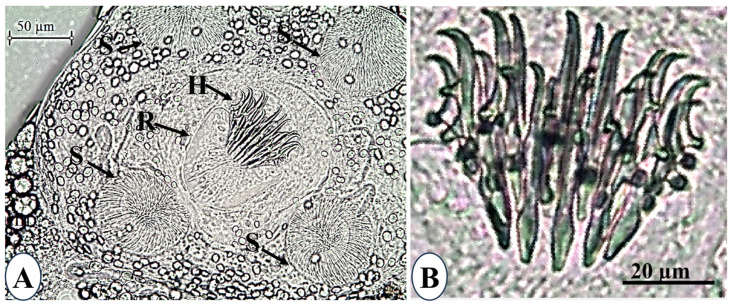
*Amirthalingamia macracantha* larval stage isolated from the intestine of *Oreochromis niloticus*. (**A**) Shows a magnified view of the scolex, rostellum (R), hooks (H), and four suckers (S); (**B**) shows a magnified view of the hooks.

The neck of the parasite was lengthy, measuring 50.6 ± 9.9 µm in breadth and 113.9 ± 4.0 µm in length (Figure 4 and Figure 5A), with a subspherical scolex having a diameter of 314.94 ± 4.8 µm (Figure 4 and Figure 5), bearing four subspherical suckers, each measuring 73.93 ± 0.95 µm (Figure 6A).

Additionally, the rostellum featured a double crown with two rows of 20 hooks arranged in a subspherical shape, measuring 53.5 ± 14.0 µm in diameter (each row containing 10 hooks; Figure 6B).

The upper row had four large hooks measuring 49.3 ± 1 µm with a handle measuring 26.65 ± 0.36 µm and a curved blade measuring 24.34 ± 0.42 µm. The blade-to-handle ratio was 1:1.1. In addition, there were six tiny hooks measuring 42.8 ± 0.6 µm, with a handle length of 24.48 ± 0.19 µm, and a curved blade length of 21.52 ± 0.43 µm, with a blade-to-handle ratio of 1:1.1. The hooks in the lower row were almost identical in length (26.21 ± 0.13 µm) and smaller than those in the upper row. Their curved blade length (13.66 ± 0.3 µm) and handle length (13.25 ± 0.4 µm) barely differed. The blade-to-handle ratio was 1.03:1. The encysted larval stage in this study was morphologically and morphometrically compared with other encysted larvae (Table 1).

#### Parasite Movement Description

**Figure 7:**
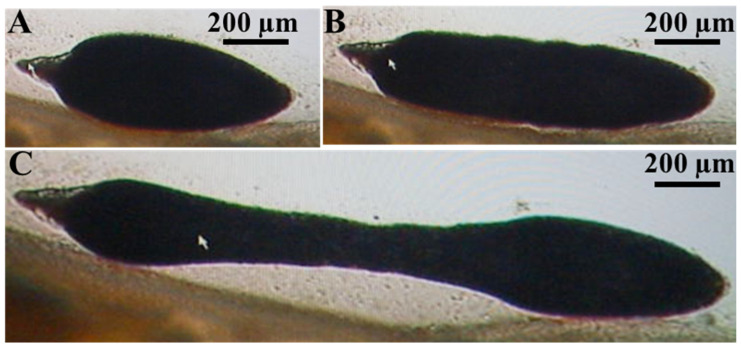
Movement of *Amirthalingamia macracantha* larval stage involves shrinking toward its middle (**A**) and then extending longitudinally towards the outer sides with mechanical movements, (**B**) and (**C**). These images were taken from Supplementary Materials Video S1.

The larval stage contracts longitudinally towards its center (Figure 7A) and then fully extends its body (Figure 7B,C, Supplementary Materials Video S1).

### 3.2. Histopathology

**Figure 8:**
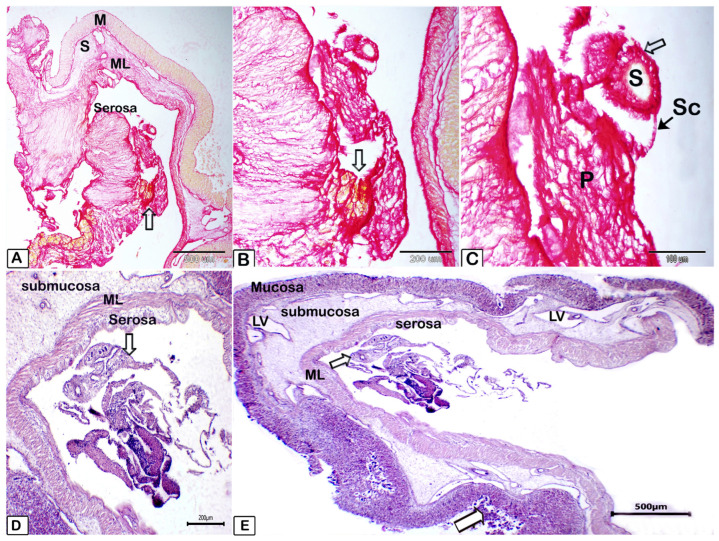
Illustrating micrograph of paraffin sections of stomach showing *Amirthalingamia macracantha* larval stage attached to the stomach utilizing Picrosirius red (**A**–**C**) and hematoxylin and eosin stains (**D**,**E**). (**A**–**C**): various magnifications illustrating that *Amirthalingamia macracantha* larval stage was located both free and affixed to the serosal layer. *Amirthalingamia macracantha* larval stage (P) exhibiting the scolex (sc, black arrow) and sucker (S) with spinitriches (white arrow). (**D**,**E**): *A. macracantha* larval stage shown by the white arrow. A large white arrow indicates the stomach mucosa epithelium that had sloughed off. The stomach consists of four layers: mucosa (m), submucosa (sub), muscular layer (ML), and serosa (S). Lymphatic vessels exhibiting dilatations (LVs).

**Figure 9:**
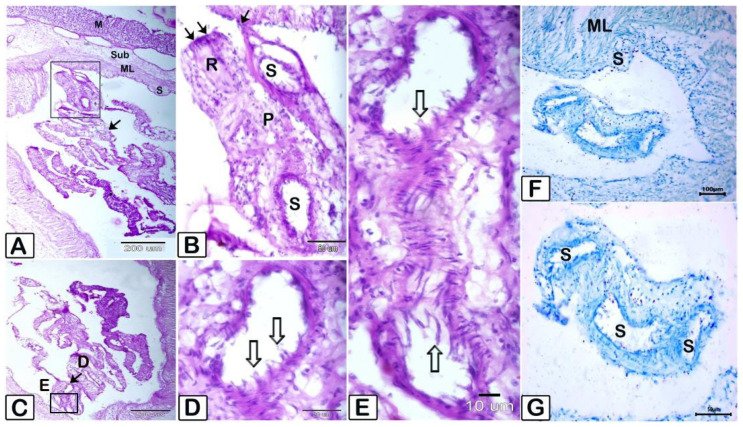
Micrograph of paraffin sections of stomach displaying the attachment of *Amirthalingamia macracantha* larval stage to the stomach and the morphology of the rostellum and suckers using various staining techniques. (**A**–**E**) stained with PAS/Hx, (**F**,**G**) stained with methylene blue (**A**), *Amirthalingamia macracantha* larval stage (shown by arrows) apparent, adhered to the serosal layer of the stomach. (**B**) Higher magnification of the square region from (**A**), representing a larval stage (P) with the rostellum (R) bearing hooks (double black arrow). One sucker (S) is affixed to the host tissue (black arrow). (**C**–**E**) *A. macracantha larval stage* sucker (black arrow) with spinitriches (white arrows). (**F**,**G**) *A. macracantha larval stage* suckers (S) stained with methylene blue. The stomach comprises four layers: mucosa (m), submucosa (sub), muscle layer (ML), and serosa (S).

**Figure 10:**
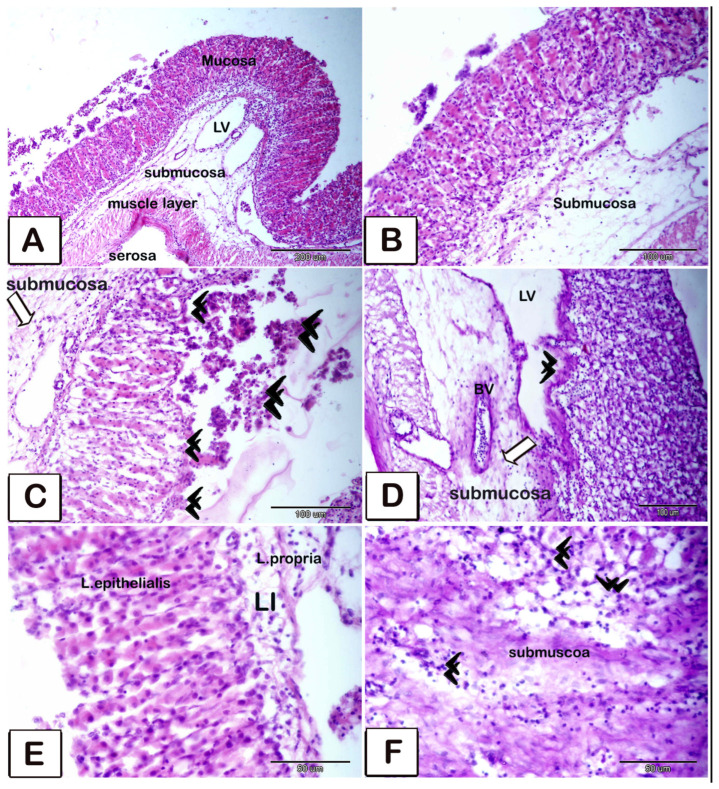
Paraffin sections of the stomach stained with hematoxylin and eosin (**A**–**C**,**E**) and periodic acid–Schiff with hematoxylin (**D**,**F**) to illustrate the histological alterations. (**A**,**B**) Overview of the stomach illustrating that it consists of four layers: mucosa (m), submucosa (sub), muscular layer (ML), and serosa (S). Observe the dilation of lymphatic vessels adjacent to the thickened wall (double arrowheads). (**C**,**D**) Illustration of the erosion of gastric epithelium (double arrowheads in figure (**C**)) and submucosal layer, accompanied by a significant influx of inflammatory cells (white arrows) into the blood vessels (BVs). Observe the dilation of lymphatic arteries adjacent to the thickening of the wall (double arrowheads in figure (**D**). (**E**,**F**): Many inflammatory cells (double black arrowheads) present in the lamina propria and submucosa layers. Particularly, lymphocytes of varying sizes within the lamina propria beneath the epithelial lamina.

**Figure 11:**
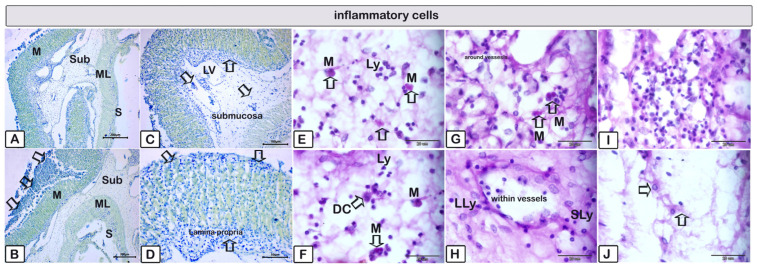
Paraffin sections of the stomach stained with methylene blue (**A**–**D**) and PAS-HX (**E**–**H**) to illustrate inflammatory cells throughout various layers. (**A**,**B**) Overview of the stomach illustrating that it consists of four layers: mucosa (m), submucosa (sub), muscular layer (ML), and serosa (S). Observe the desquamated epithelial layer of the gastric mucosa (arrows). (**D**,**E**) Inflammatory cells within the lamina propria layer (arrows). (**E**–**H**) Higher magnifications of inflammatory cells within the submucosal layer. Numerous lymphocytes of varying sizes, both large (LLy) and small (SLy), alongside mast cells (Ms, indicated by arrows) releasing their contents and undergoing degranulation; dendritic cells (Ds, indicated by arrows) were identified among and inside the vasculature, indicative of inflammatory cell presence. (**I**,**J**) Plenty of inflammatory cells were observed within the submucosal layer, with dendritic cells identified by pointed arrows.

**Figure 12:**
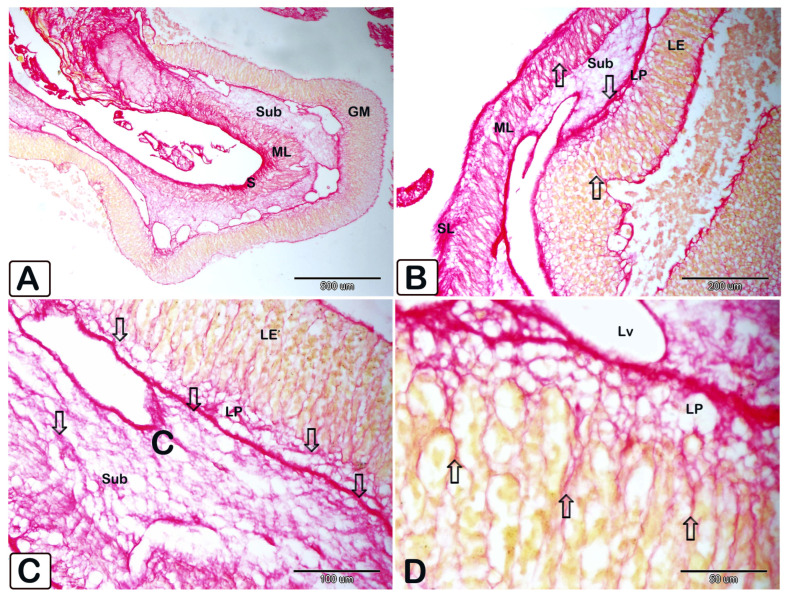
Paraffin sections of the stomach stained with Picrosirius red to illustrate red fibrous tissue throughout various layers. (**A**–**D**) Overview of the stomach illustrating that it consists of four layers: mucosa (m), submucosa (sub), muscular layer (ML), and serosa (S). Various magnifications illustrate the red fibrous tissue (arrows) throughout various layers. The stomach comprises four layers: mucosa (m), submucosa (sub), muscle layer (ML), and serosa (S).

**Figure 13:**
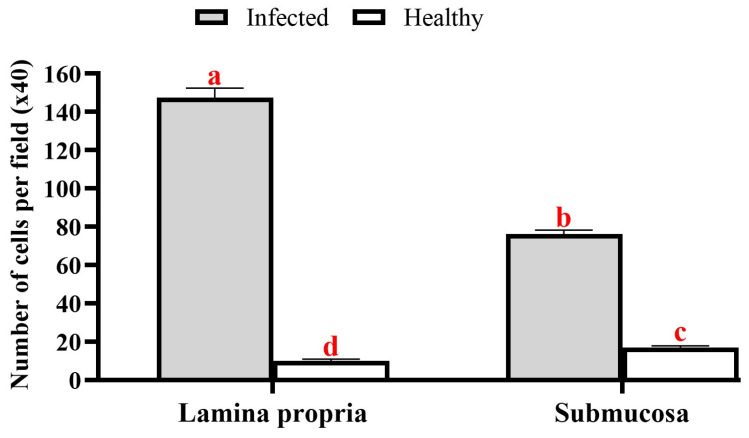
Quantitative investigation of the inflammatory cell counts in the lamina propria and submucosa layers of the infected fish stomach with *Amirthalingamia macracantha* larvae. Using two-way ANOVA (Tukey test), different letters showed a significant difference.

*A. macracantha* larval stage was observed attached within the serosal layer of the stomach of *O. niloticus*. The morphology of the rostellum and suckers was examined using various staining procedures, such as Picrosirius red, hematoxylin and eosin stains, PAS (periodic acid–Schiff)–Harris hematoxylin, and methylene blue. The larval stage features a rostellum supported by hooks and equipped with spinitriches (Figure 8 and Figure 9). These parasites seem to irritate and damage the surrounding tissues, causing loss of gastric epithelium, enlargement of lymph vessels in the submucosal layer, and an increase in inflammatory cells in the stomach’s lamina propria, submucosa, and blood vessels. This includes mast cells, lymphocytes of different sizes, and dendritic cells. Figure 10 and Figure 11 show degranulating mast cells. Figure 12 displays the abundant red fibrous tissue present in various layers of the stomach. Figure 13 shows the number of inflammatory cells in the submucosa (76.1 ± 4.7; CI 95% 71.5–80.8) and lamina propria (144.4 ± 10.3; CI 95% 134.1–154.7), which was significantly elevated when they were compared to the control group in both the submucosa (17.1 ± 2.2 (CI 95% 14.9–19.3) and lamina propria (10.3 ± 1.2 (CI 95% 9.0–11.6). The average percentage of fibrosis was 11.9 ± 1.1 (CI 95% 10.9–13.0).

## 4. Discussion

**Figure 14:**
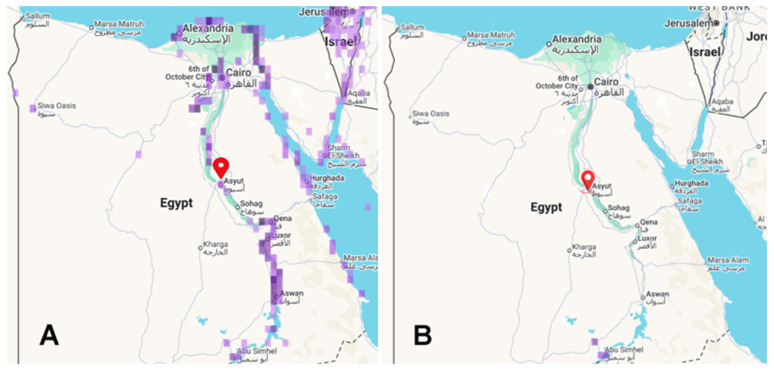
Spread of cormorant across Egypt based on eBird data: (**A**) great cormorant, *Phalacrocorax carbo* (https://ebird.org/species/grecor/EG-ASN, accessed on 31 July 2025), and (**B**) reed cormorant, *Microcarbo africanus* (https://ebird.org/species/lotcor1, accessed on 31 July 2025).

**Figure 15:**
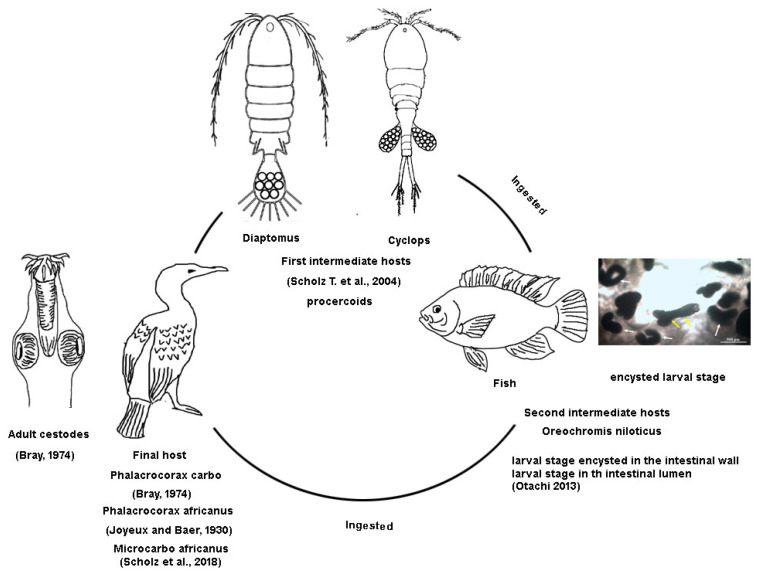
Illustration demonstrating the potential life cycle of *Amirthalingamia macracantha*. The figure was created by Dr. Yosra M. I. El Sherry using Photoshop software (v21.0.6), images taken during this study, and literature images.

Cestodes are vertebrate endoparasites that complete their life cycle in their definitive hosts, which carry the adult stage, after requiring at least one intermediate host [16]. An adult *A. macracantha* was found in the intestinal tract of two cormorants: a long-tailed cormorant (*Microcarbo africanus*) in Mali and a great cormorant (*Phalacrocorax carbo*) in Sudan [11,15]. Initially, the adult was first identified by Joyeux and Baer [38] in a reed cormorant (*Phalacrocorax africanus*) in the Niger Valley, Mali, as *Dilepis delachauxi* [39]. It was later renamed *Paradilepis delachauxi* by Joyeux and Baer [40]. Subsequently, according to Bray [15], their 1930 specimens were renamed *Paradilepis macracantha*. Figure 14 illustrates the spread of the great cormorant, *Phalacrocorax carbo*, and the reed cormorant, *Microcarbo africanus*, across Egypt. Figure 15 illustrates the putative life cycle of *A. macracantha*.

Fish have been shown to act as second intermediate hosts for cestodes by ingesting infected crustaceans such as Diaptomus or Cyclops [11,12,13]. Fish can be infected by *A. macracantha* through passive transfer [13]. The parasite has been detected in *O. niloticus* populations in Uganda, Ethiopia, Sudan, and Kenya [5,13,14,15,16,17], as well as in subspecies of *O. niloticus* such as *O. niloticus baringoensis* [41] and *O. niloticus leucostictus*. It has also been observed in redbelly tilapia (*Tilapia zillii*) [14,42], as well as in cyprinid fish species like common carp (*Cyprinus carpio*) and straightfin barb (*Barbus paludinosus*) in Kenya [42]. Furthermore, it has been documented in Zambezi bream (*Pharyngochromis acuticeps*) in Zimbabwe, banded tilapia (*Tilapia sparrmanii*), and southern mouth brooders (*Pseudocrenilabrus philander*) [43,44,45]. There were only two studies [45,46] on African catfish (*Clarias gariepinus*) in Nigeria and Congo. The frequent reports of its presence alongside *O. niloticus* may be attributed to its diet, as it primarily feeds on benthic invertebrates, zooplankton, phytoplankton, and macrophytes [47,48,49]. Many parasites utilize benthic and zooplankton species as intermediate hosts for their life cycle [50].

In this investigation, 35 per cent of the *O. niloticus* samples were found to have tiny spherical red nodules representing the metacestode larval stage in the anterior and posterior portions of the intestine and stomach, respectively. These findings were also reported by Florio and colleagues [16]. The parasite cysts could only be detected in the stomach and intestinal wall in the present study. However, the encysted larval stage was found in different locations among different fish species. According to reports by Akoll et al., 2012 [50], and Akoll et al., 2012 [13], specimens of *O. niloticus* obtained from Uganda had larval stages confined to their intestines. Additionally, Adamba and colleagues [41] revealed that encysted *A. macracantha* larval stages were highly prevalent (89.62 per cent) along the intestinal wall of *O. niloticus baringoensis* in Kenya. Moreover, Aloo [14] reported that 10.7% of *T. zillii* cases in Kenya also had cysticercoids encysted in their intestine. Furthermore, the intestines of *B. paludinosus* (3%), *T. zillii* (3.2%), and *O. leucostictus* (21.4%) in Lake Naivasha Kenya, as well as the intestine and liver of *T. zillii* (79.5%) in Lake Turkana, Kenya, were reported to harbor *A. macracantha* [36]. Nevertheless, it was detected in the liver of 2.7% [17,51] and 3.3% [51] of *O. niloticus* samples investigated in Ethiopia and in both the liver and the intestinal wall and its lumen of *Tilapia nilotica* in Sudan [15]. Moreover, larvae have been found in the intestinal wall, liver, and body cavity of *T. zillii* [45]. The presence of larvae in the liver or body cavity may be explained by the migration of some cestodes’ larval stages into parenteral locations to protect themselves from being shed from the host intestine. This is particularly common in cold weather when nutrition is severely reduced, as seen in the case of *Proteocephalus ambloplitis* larval stages. However, when the water temperature increases, the parasite reinfects the intestine, where it matures into an adult worm [52,53]. In the present study, the observed larval metacestode stage was the larval stage, either encysted, free in the intestinal lumen, or attached to the intestinal serosa. This suggests that *O. niloticus* acts as a second intermediate host for this parasite. Otachi [42] obtained similar results, revealing for the first time that *T. zillii* has two metacestode life stages: an encysted larval stage in the intestinal wall and non-encysted larval stages. Global warming is an important factor influencing the presence of multiple larval stages within a single host. The excystation of metacestodes, as they move from intermediate fish hosts to fish-eating birds, is triggered by the higher water temperatures. Thus, he postulated that *A. macracantha* within *T. zillii* might develop into worms that mature into premature adult worms [42].

Variations in water parameters may lead to fluctuations in infection prevalence. According to Aloo [14], the lower prevalence of *Amirthalingamia* sp. in *T. zillii* in Oloidien Bay, Kenya, is due to the intermediate host’s inability to tolerate high water salinity. The presence of the parasite is positively correlated with temperature, pH, electrical conductivity (EC), nitrogenous compounds (NH_4_, NO_2_, NO_3_, and TN), turbidity, soluble reactive phosphorus, and nutrient levels [5,41]. The water’s electrical conductivity and nutrient levels contribute to an increase in phytoplankton production, leading to higher concentrations of chlorophyll a that enrich the food chain. This increase in the phytoplankton population leads to a higher population of intermediate hosts and, as a result, more parasite infection [54,55]. Additionally, poor water quality caused by high nutrient levels can stress fish and make them more vulnerable to parasitic infections [55,56].

The number of metacestodes per fish ranged from 1 to 45, with a mean intensity and abundance of 10 ± 1.7 (CI 95% 8.2–11.7) and 3.5 ± 0.05 (CI 95% 3.4–3.5), respectively. However, Akoll and colleagues [50] found 11 parasites per fish with a mean intensity of 11 and a mean abundance of 4.9. Meanwhile, Adamba et al. [41] reported a mean intensity of 46.37 and a mean abundance of 41.56. Otachi [42] found that the mean intensities in *T. zillii*, *B. paludinosus*, and *O. leucostictus* were 27, 9.5, and 8.1, respectively.

The K factor is used to compare the condition of fish based on the concept that larger fish of a certain length have better physiological conditions [57]. It also serves as a helpful indicator for monitoring the age, growth rate intensity, and feeding habits of fish [58]. According to Olurin and Aderibigbe [59], fish exhibit isometric growth when their length increases in proportion to their body weight. A regression coefficient of “3” indicates isometric growth, while values greater than “3” indicate allometric growth. The estimated K factor in the current study was 1.67 ± 0.03 (CI 95% 1.64–1.69), suggesting that the encysted metacestodes hurt the infected *O. niloticus*.

The metacestode body was elongated and folded with a subspherical scolex and four subspherical suckers. The subspherical rostellum was everted and carried paired crowns, each with ten hooks. This current description aligns with the previous ones of Bray [15] and Uhrová [10], who described the metacestode as having an oval, elongated, and folded body; an evaginated scolex with four round suckers; and a stretched or retracted rostellum bearing 20 hooks of three different sizes divided into two rows. Comparative measurements are listed in Table 1.

In the present study, larval length ranged from 1228.5 ± 34.7 µm, while previous research has reported sizes up to 5000 µm [10]. This variation in larval size is likely influenced by the complex life cycles of helminths, such as cestodes, which typically require two hosts: an intermediate host for the larval stage and a definitive host for the adult stage. Parasites tend to grow more extensively in a host that offers a safer and faster growth environment. When the mortality risk is high in a host, the parasite may limit its development and transition earlier to the next host. Generally, larger hosts provide more resources and space, which can support greater parasite growth. This may be due to the host’s capacity to tolerate the parasite and survive longer. However, larger hosts may also possess more robust immune defenses. Interestingly, parasites that infect large intermediate hosts are often found in large definitive hosts, possibly because large predators tend to feed on larger prey. Some parasite species may experience growth trade-offs between hosts; enhanced growth in one host could limit growth in the other. However, in many cases, growth appears to occur independently in each host species [60]. In the current study, the examined infected fish had a mean weight of 33.5 ± 6.7 g, a total length of 12.5 ± 0.7 cm, and a standard length of 10.0 ± 0.7 cm. Unfortunately, body measurements of fish were not reported in the earlier studies referenced in the comparative table. This lack of data prevents a direct comparison of the relationship between fish size and larval development across studies.

Helminths generally cause chronic infections by migrating through the host’s tissues, frequently resulting in significant and long-term problems. Parasite infection results in two forms of tissue damage in the host: direct damage inflicted by the pathogen and subsequent injury resulting from the immune response (immunopathology; [61]). We observed a profusion of lymphocytes beside mast cells demonstrating degranulation. The previous research by Abdel Hakeem et al. [37] agrees with our findings about the significant presence of degranulated mast cells in parasite infections. For the host to fight off parasite infestations, the degranulation of mast cells is essential. Parasitic helminth infections are well known to involve MCs [62]. When activated, MCs release enzymes like chymase, tryptase, and carboxypeptidase A from cytoplasmic granules that store a variety of prepared mediators like histamine and heparin [63]. Reite [64] indicated that fish mast cells exhibit similarities to mammalian mast cells. Researchers have seen mast cell degranulation, cytokine release, and the consequent inflammatory response in teleost fish due to infection [65].

The release of histamine and other vasoactive mediators enhances vascular permeability and local blood flow while also stimulating smooth muscle to facilitate the expulsion of mucosal parasites. Furthermore, histamine promotes the production of mucus by epithelial cells, potentially facilitating pathogen immobilization and cyto-protection. Mast cells produce chemotactic factors that enhance the recruitment of various inflammatory cells, including eosinophils (eotaxin), natural killer (NK) cells (IL-8), and neutrophils (IL-8 and TNF-α) [66,67].

Mast-cell-derived cytokines and chemokines facilitate the migration of dendritic cells (DCs; TNF-α and CCL20) and effector T cells (CXCL10/IP10 and CCL5/RANTES) to the infection site and draining lymph nodes. Mast cells can serve as antigen-presenting cells, especially for CD8+ T cells. Mast cell products can enhance the maturation of immature dendritic cells, as well as upregulate antigen presentation and the expression of co-stimulatory molecules. Mast-cell-derived histamine promotes the polarization of naive T cells towards a Th2 phenotype by decreasing DC production of IL-12 and enhancing IL-10 secretion in response to LPS [68].

Our study demonstrated the degranulation of mast cells accompanied by fibrosis. There is a relationship between the MC count and the duration of disease, as in cutaneous leishmaniasis in Leishmania infections. Infected animals show a rise in MC number and activation during *T. gondii* infection. In cases of *T. cruzi* infection, the colon musculature of chronic Chagas patients with megacolons or the myocardium of patients with Chagas cardiopathy exhibit higher MC numbers and increased fibrosis. There was a notable increase in the influx of neutrophils and macrophages towards the peritoneal cavity, and the number of degranulated MCs was substantially larger than that of intact MCs [63].

Infections by *A. macracantha* pose a significant threat to aquaculture due to their ability to damage host tissues and induce inflammatory responses. In this study, the number of metacestodes per infected *O. niloticus* ranged from 1 to 45, with infected fish showing a decreased condition factor (1.67 ± 0.03) compared to their uninfected counterparts (2.1 ± 0.05). This highlights the negative impact of parasites on fish health and aquaculture productivity. *Gryporhynchid larvae* can disrupt the normal growth and weight development of fish due to harmful pathological changes in the fish’s internal organs. Infection by *gryporhynchid* larvae, specifically *Valipora campylancristrota*, only caused damage to the gallbladder and other internal organs in fish when parasite loads were high, typically ranging from dozens to hundreds. Fish with severe infestations exhibited significantly slower growth and lower body weight compared to those with light or no infections [11].

The presence of conspicuous red cysts caused by *A. macracantha*, a member of the Gryporhynchidae family, in the stomach and intestines of *O. niloticus* diminishes the visual quality of the fish. This makes them less desirable to both consumers and processing companies. As a result, these fish are often rejected or sold at reduced prices, leading to economic losses [69].

## 5. Conclusions

The larval stage of *A. macracantha*, Gryporhynchidae (Cestoda: *Cyclophyllidea*) were discovered for the first time in Assiut City, Egypt. They appeared as red nodules on the mucosal surface of the stomach and intestine of wild *O. niloticus*. The prevalence of these metacestodes was notably high (35 per cent), with a mean intensity of 10 ± 1.7 (CI 95% 8.2–11.7) and a mean abundance of 3.5 ± 0.05 (CI 95% 3.4–3.5). The pathological impact of these metacestodes on the afflicted host tissue was recognized, along with the morphological characteristics of the larval stage. However, further studies should be conducted to clarify the impact of *A. macracantha* on the fish’s immune system at the gene level and to complete its life cycle in its final host. Additionally, future research could investigate the impact of environmental factors such as climate change and water quality on *A. macracantha* infections.

## Figures and Tables

**Table 1:** Comparative measurements (µm) of the *Amirthalingamia macracantha* metacestode from this study with the previously described studies.

Character				
**Reference**	Current study	Bray [15]	Scholz, Bray, Kuchta and Řepová [11]	Uhrová [10]
**Host**	*Oreochromis niloticus*	*Tilapia nilotica*		*Haplochromis acidens* *Pseudocrenilabrus philander* *Tilapia sparrmanii*
**Country**	Egypt	Sudan		Zimbabwe
**Infected organ**	stomach & intestine	Liver		Liver
**Metacestode length**	1228.5 ± 34.7 µm	2600–4200 µm		3000–5000 µm
**Metacestode width**	312.6 ± 36 µm	1300–1800 µm		
**Scolex diameter**	314.94 ± 4.8 µm	850–1120 µm		
**Suckers’ diameter**	73.93 ± 0.95 µm	290–360 µm		
**Lower row** **Total length**	26.21 ± 0.13 µm Handle (H) 13.25 ± 0.4 µm Blade (B) 13.66 ± 0.03 µm B/H ratio 1.03: 1	260–280 µm	240–290 µm Handle 144–160 µm Blade 157–184 µm B/H ratio 0.98–1.11	258–270 µm Handle 130–145 µm Blade 150–172 µm B/H ratio 1.15–1.2
**Upper row** **Total length**	4 hooks (49.3 ± 1 µm) Handle 26.65 ± 0.36 µm Blade 24.43 ± 0.42 µm B/H ratio 1:1.1	4 hooks (450–455 µm)	4 hooks (448–480 µm) Handle 240–296 Blade 272–296 B/H ratio 1.00–1.16	4 large hooks (465–475 µm) Handle 230–280 Blade 170–280 B/H ratio 1.00–1.18
	6 hooks (42.8 ± 0.6 µm) Handle 24.48 ± 0.19 µm Blade 21.52 ± 0.43 µm B/H ratio 1:1.1	6 hooks (400–430 µm)	6 hooks (390–450 µm) Handle 224–256 µm Blade 140–280 µm B/H ratio 1.00–1.18	6 small hooks (420–430 µm)

## Data Availability

Data and materials are available upon reasonable request from the corresponding author. In addition, all data generated or analyzed during this study are included in this published article.

## Supplementary Materials

The following supporting information can be downloaded at https://www.mdpi.com/article/10.3390/ani15162334/s1, Video S1: Live images recorded under the microscope during this study showing the movement of the free stage of the encysted metacestodes.
